# A novel functional polymorphism of *GSTM3* reduces clear cell renal cell carcinoma risk through enhancing its expression by interfering miR‐556 binding

**DOI:** 10.1111/jcmm.13528

**Published:** 2018-03-22

**Authors:** Ying Wang, Zi‐Ying Yang, Yi‐Huan Chen, Feng Li, Han Shen, You Yu, Hao‐Yue Huang, Zhen‐Ya Shen

**Affiliations:** ^1^ Department of Cardiovascular Surgery First Affiliated Hospital & Institute for Cardiovascular Science Soochow University Suzhou Jiangsu China; ^2^ Department of Urinary Surgery First Affiliated Hospital of Soochow University Suzhou Jiangsu China

**Keywords:** clear cell renal cell carcinoma, glutathione‐S‐transferase Mu3, invasion, miRNA, polymorphism

## Abstract

Dysregulation of glutathione‐S‐transferase M3 (*GSTM3*) has been related to clear cell renal cell carcinoma (ccRCC) in our former study. *GSTM3* plays a pivotal role of detoxification and clearance of reactive oxygen species (ROS) in tumour tissues. This study aimed to examine: (1) the associations between *GSTM3* single nucleotide polymorphisms (SNPs) and risk of ccRCC, and (2) the potential molecular mechanism accounting for its effects. 5 SNPs in 3′UTR of *GSTM3* were initially genotyped in 329 cases and 420 healthy controls. A SNP‐rs1055259 was found to be significantly associated with the susceptibility of ccRCC (OR = 0.59, 95% CI = 0.41‐0.92; *P *=* *.019). The minor allele of rs1055259 (G allele) was associated with RCC risk. This SNP was predicted to affect microRNA (miR)‐556 binding to 3′UTR of *GSTM3* mRNA. To determine the functional impact, plasmid constructs carrying different alleles of rs1055259 were created. Compared to rs1055259 A‐allele constructs, cells transfected with rs1055259 G‐allele construct had higher transcriptional activity and were less responsive to miR‐556 changes and gene expression. Elevated *GSTM3* expression in G‐allele cells was associated with ROS activity and ccRCC development. Taken together, this study indicated that a functional polymorphism of *GSTM3* ‐rs1055259 reduced susceptibility of RCC in the Chinese population. It influenced *GSTM3* protein synthesis by interfering miR‐556 binding, subsequently suppressed ROS activity and ccRCC progression.

## 1 INTRODUCTION

Renal cell carcinoma (RCC) is a kind of highly heterogeneous tumours. Clear cell renal cell carcinoma (ccRCC) is the major histological subtype (~80%). The incidence of RCC continued to rise over the past 2 decades.^1^ It is 2‐fold higher in men than in women. Surgical resection remains the main curative treatment. In the acknowledge of RCC therapy, it does not respond to radiotherapy and chemotherapy effectively, but responds to targeted therapies partially. Although the application of targeted therapies for RCC is promising, but there are many problems of these novel strategies.^2^ Little is known about the advancement of specific prognostic or risk molecular markers for RCC.

Recent genome‐wide association studies (GWAS) indicated that several loci mapped on 2p21, 2q22.3, 11q13.3, 12p11.23 and 12q24.31 were associated with RCC risk significantly.^3^, ^4^, ^5^ However, the genetic risk factor identified in Caucasian population is not so consistent with that in Asian population.^6^ The causal genetic factors for RCC are still unclear.

In our previous study, we have screened the differentiated genes between metastasis and primary ccRCC cells using cDNA microarray. Glutathione‐S‐transferase Mu3 (*GSTM3*), a *GSTM*‐class subunit, was identified as a notably down‐regulated gene. It both down‐regulated in metastatic vs primary ccRCC cells and primary ccRCC cells vs adjacent normal renal tissues.^7^ We also studied the tumour suppressor role of *GSTM3* in the progression of ccRCC and a polymorphism rs1332018 which was significantly associated with the postoperative prognosis of ccRCC.^8^

In the current study, we report a novel SNP in ccRCC‐ rs1055259 in the 3′untranslated region (UTR) of *GSTM3* significantly reduced the ccRCC risk and influence the binding of miR‐556 to the 3′UTR of *GSTM3*. This might be the potential mechanism of rs1055259 impact the renal carcinoma risk.

## 2 MATERIALS AND METHODS

### 2.1 Study population and postoperative Follow‐up

Consecutive ccRCC patients who received curative nephrectomy at Department of Urinary Surgery in Changzhou first people's hospital, Zhenjiang first people's hospital and the first affiliated hospital of Soochow university from May 2006 to November 2015. Healthy controls were randomly selected from people who received physical examination at Advanced Physical Examination Center in the first affiliated hospital of Soochow University. The demographics and clinical features of the ccRCC patients and controls involved in the study are listed in Table 1. Cases and controls were frequency matched on gender and healthy controls were older than patients. All the patients were pathologically confirmed and signed the informed consent. Follow‐up was performed via in person interview or telephone calls according to the standard epidemiologic procedure twice a year. The final date of follow‐up was 12 December 2015. All the protocols conformed to the 1975 Declaration of Helsinki and were approved by ethics committee of the first affiliated hospital of Soochow University.

**Table 1:** Demographic and clinical features of the study subjects

Characteristic	Case‐control study		
	RCC cases (%) N = 329	Controls (%) N = 420	*P*
Age (y)			
Average (Range)	55.62 (20‐85)	60.97 (24‐93)	**<.001**
≤40	41 (12.46)	32 (7.62)	–
40‐60	179 (54.41)	206 (49.05)	–
60‐80	102 (31.00)	123 (29.28)	–
>80	7 (2.13)	59 (14.05)	–
Gender			
Male	226 (68.69)	286 (68.10)	.861
Female	103 (31.31)	134 (31.90)	–
Histology			
Clear cell	329 (100%)	–	–
Papillary	0 (0)	–	–
Chromophobe	0 (0)	–	–
AJCC stage			
I	247 (75.08)	–	–
II	49 (14.89)	–	–
III	23 (7.00)	–	–
IV	10 (3.03)	–	–

AJCC, American Joint Committee on Cancer; RCC, renal cell carcinoma. The significant *P* value was shown in bold.

### 2.2 Blood samples collection and ccRCC cells primary culture

Ethylene diamine tetraacetic acid (EDTA) anticoagulated peripheral blood samples were collected from patients before nephrectomy and healthy controls. Genomic DNA was extracted using a RelaxGene Blood DNA System (TIANGEN biotech, Beijing, China) according to the manufacturer's instruction. The fresh surgical specimens of ccRCC from 17 patients were harvested and immediately immersed in ice‐cold PBS containing 1% penicillin/streptomycin and 0.5% glutamine (Beyotime, Shanghai, China). Primary cell culture was performed within 60 min after surgery as previously described.^7^ As a variation of phenotype might occur at higher passages, we chose the ccRCC cultures at the fourth passage for functional experiments.

### 2.3 SNP selecting and genotyping

**Figure 1:**
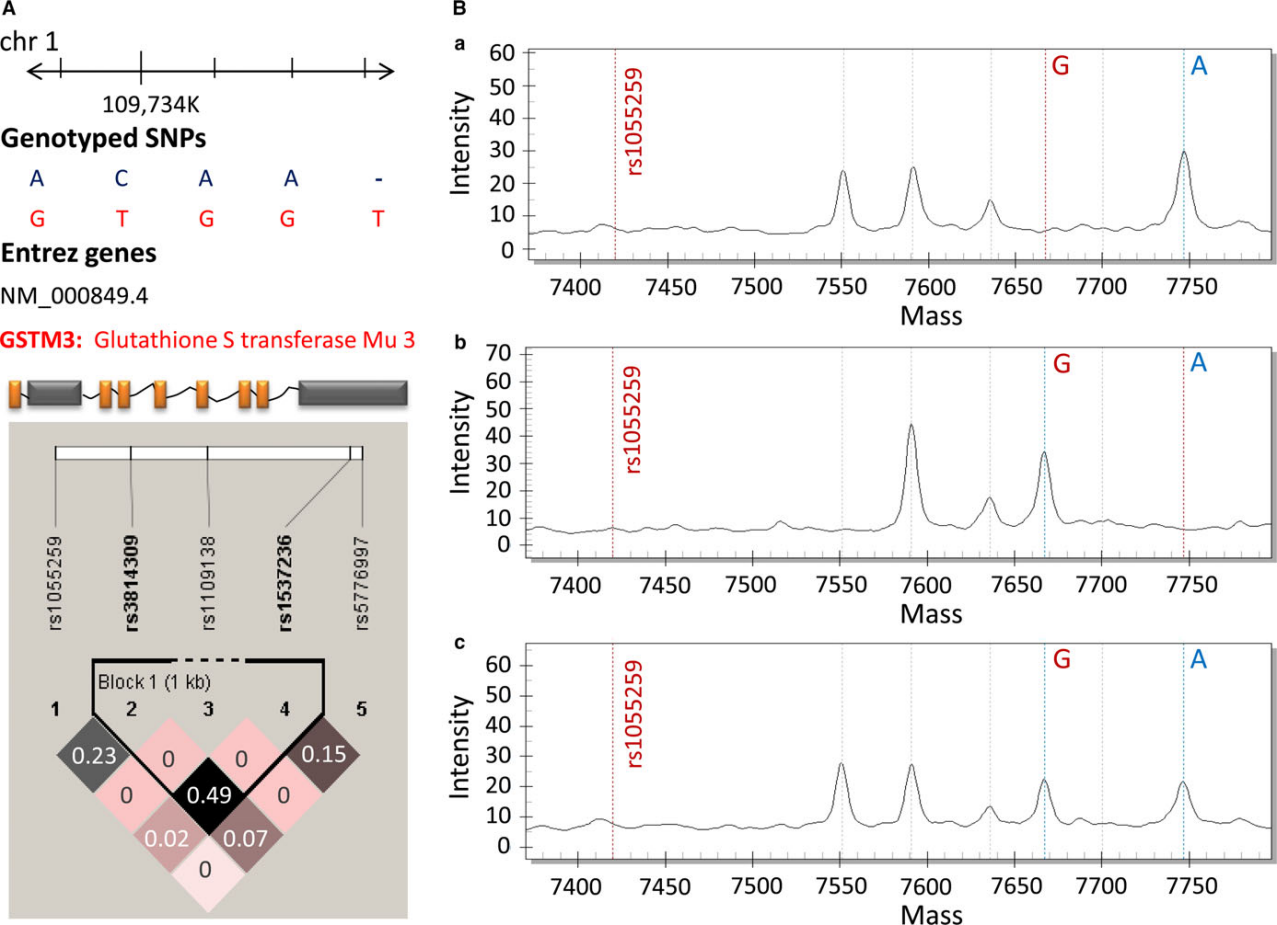
The linkage disequilibrium (LD) plot of SNPs in *GSTM3* gene and representative MALDI‐TOF MS spectra of rs1055259. (A) Pairwise LD among 5 candidate SNPs of *GSTM3*. The r2 value is shown in each diamond. It indicates the pairwise correlation between SNPs. The colour scale ranges white to black reflect lower to higher r2 values. (B) Genotypes of rs1055259 are confirmed by plotting peak intensity (*y*‐axis) against mass (Da) (*x*‐axis). (a) MALDI‐TOF MS spectrum of a single peak at 7748.5 Da represents homozygous genotype of AA. (b) MALDI‐TOF MS spectrum of a single peak at 7667.0 Da indicates homozygous genotype of GG. (c) MALDI‐TOF MS spectrum of 2 peaks at 7748.5 Da and 7667.0 Da indicates heterozygous genotype of AG

We selected 5 SNPs by analysing *GSTM*3‐related Han Chinese data from 1000 Genome Project resources (http://www.1000genomes. org). The candidate SNPs should meet these criteria: (1) the minor allele frequency (MAF) > 0.05; (2) *r* ^2^ < .80 (Figure 1A); (3) at the 3′UTR of *GSTM3*. The significant SNP rs1055259 is located at a miR‐556 binding site, with predicted proximal transcriptional regulatory potential (http://rsnp.psych.ac.cn/) (http://snpinfo.niehs.nih.gov/cgi-bin/snpinfo/ snpfunc.cgi). rs1537236, rs3814309, rs1109138 and rs5776997 were also predicted as functional SNPs, which seated in other miRNA binding sites.

All SNPs were genotyped using matrix‐assisted laser desorption ionization time‐of‐flight mass spectrometry (MALDI‐TOF MS) and analysed on the MassARRAY platform (Sequenom, San Diego, CA, USA). The MALDI‐TOF MS spectra of rs1055259 were shown in Figure 1B. Amplification and single‐base extension primers applied in multiple PCR were synthesized by Benegene (Benegene Biotechnology, Shanghai, China) (Table 2). The product of each sample was dispensed onto a 384‐format SpectroCHIP with the MassARRAY Nanodispenser RS 1000. Then, the MALDI‐TOF MS assay was performed on a MassARRAY Compact Analyzer. Genotype calling was conducted using MassARRAY RT software version 3.0. Data were analysed with MassARRAY Typer software 4.0.3. Genotyping quality was assessed by Sanger sequencing of ~10% randomly selected samples, yielding a 100% concordance.

**Table 2:** Primers for PCR and single‐base extension reaction in MALDI‐TOF MS assay

Gene (GSTM3) Loci	Chr:position	Forward primer^a^	Reverse primer^a^	Extension primer	Amplicon size (bp)
rs1537236	1:109736350	linker‐GCCCATAGGCTGCTCTGC	linker‐ GCCTTAGGAAAAGTTGTAG	CACAAA GGAAAGATTT	121
rs3814309	1:109734781	linker‐GTGGCAAAACATGGCA GTTG	linker‐ CAGGGATACCCAGTGATGTC	TTAGCACCTTCTGTCTG	78
rs1109138	1:109735327	linker‐AGCTTCAAATTCCTGG GCTC	linker‐ ACCCCTGTCTCCCAAAAATT	GGACTATGCATGAGCAA	69
rs1055259	1:109734239	linker‐AGTGACCATCCCAACTTTGG	linker‐ CCATGGGGGTGCTAAAACCAC	TTTAACCCAGCTCATTAT	119
rs5776997	1:109736432	linker‐GGGGTAGGAGAAGTTT CTTT	linker‐ TACTCCACAGGCAGAGCAG	CTTTAAAGACGTAAAAAGAAAAAA	86

a linker = 10‐mer linker ACGTTGGATG placed at 5′‐end of each primer.

### 2.4 Bioinformatic prediction of candidate SNPs

The SNP‐flanking region RNA was online analysed using RNAfold (http://rna.tbi.univie.ac.at/cgi-bin/RNAWebSuite/RNAfold.cgi) and SNPfold (http://ribosnitch.bio.unc.edu/Downloads/SNPfold/). SNPs at the 3′UTR were predicted for putative miRNA binding site applying 3 algorithms: TargetScan (http://targetscan.org/), PicTar (http://pictar.mdc‐ berlin.de/) and miRBase (http://www.mirbase.org/search.shtml).

### 2.5 Quantitative real‐time PCR analysis of miR‐556

The expression level of miR‐556 was detected using RNA‐tailing and primer‐extension real‐time PCR according to the instructions of All‐in‐One miRNA Detection Kit (Fulengen, Guangzhou, China). miR‐556 forward primer: 5′‐AGAAGCAATTCTAGAAG‐3′; the reverse primer was miRNA universal adaptor PCR primer (Fulengen).

### 2.6 Luciferase reporter assay

**Figure 2:**
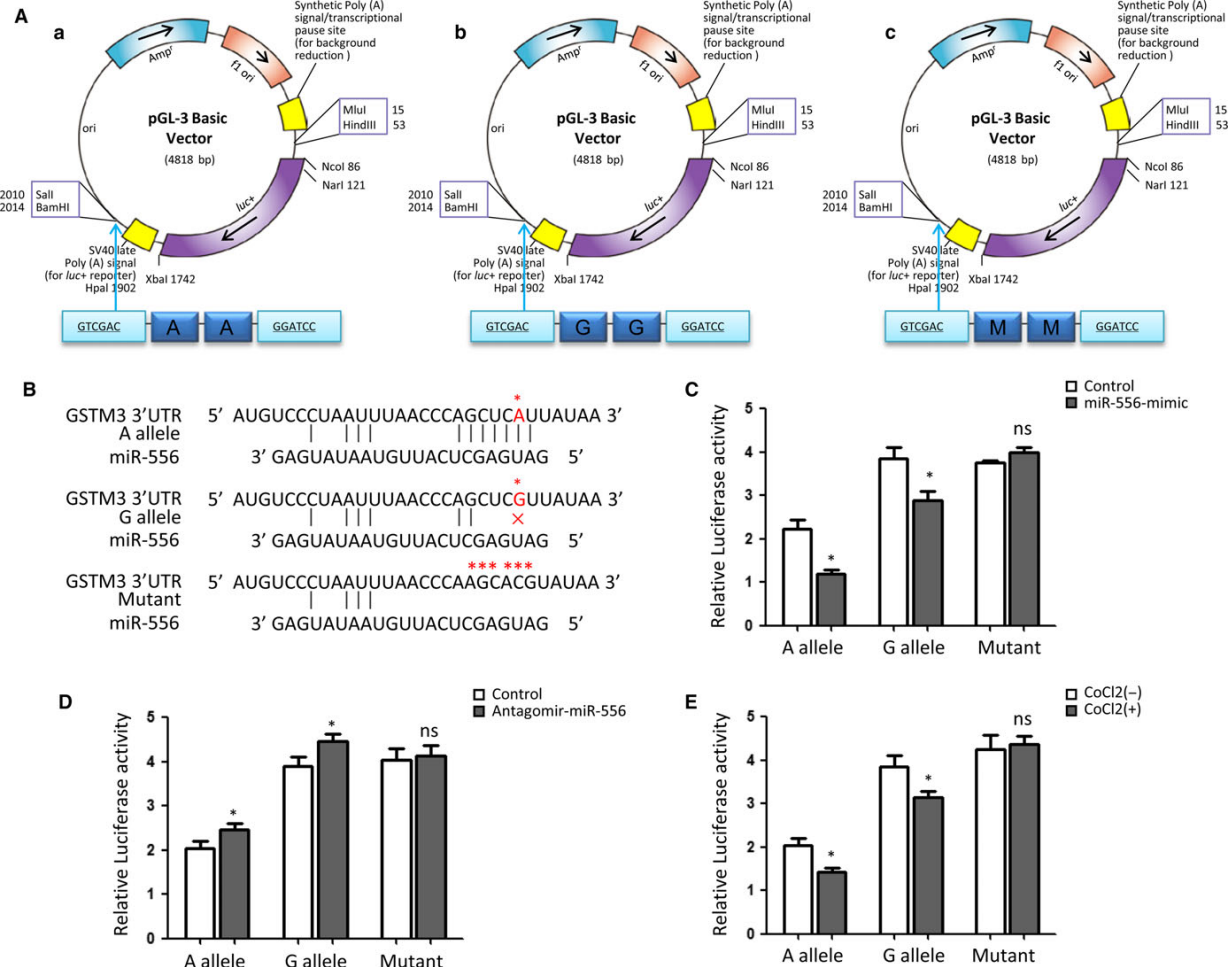
SNP rs1055259 affects luciferase activities in 786‐O cells. (A) Reporter plasmids construct. The schematic diagrams show the luciferase reporters carrying 2 copies of rs1055259‐A allele or G allele or mutant sequence at the 3′UTR of Luciferase gene. (B) This plot shows the theoretical miRNA‐mRNA duplex pairing between miR‐556 and *GSTM3* 3′UTR. The A, G alleles and mutant sequence are highlighted with an asterisk (*). (C, D) Relative luciferase activities (vs Renila luciferase) were measured in ccRCC cells expressing either A allele, G allele or mutant construct which were treated with control miR, miR‐556 mimic or antagomir‐miR‐556. (E) After incubated with 50 μmol/L CoCl2 for 24 h, luciferase activities were examined in cells transfected with either A allele, G allele or mutant construct. Six replicates for each group and the experiment were repeated at least 3 times. *P <* *.05, ns: non‐significant compared between treatment group and control group

To evaluate the binding of miR‐556 to 3′UTR of *GSTM3*, 3 reporter constructs carrying 2 copies of rs1055259‐A allele, G allele or mutant sequences at the 3′UTR of luciferase gene were created. Firstly, three ~100‐bp DNA sequences centred at rs1055259 (A or G allele) or mutant sequence were synthesized; then, 2 tandem copies of these sequences were cloned into pGL‐3 Basic vector (Promega, Madison, WI, USA) using restriction enzyme sites‐Sal I and BamH I. The schematic diagram for vector construction is shown in Figure 2A. Day 1, 786‐O cells were seeded at 1 × 10^4^ cells/well in a 24‐well plate. Day 2, cells were transfected with 0.8 μg pGL‐3 Basic vectors (inserted with rs1055259‐ A allele, G allele or mutant sequences) and 0.16 μg pRL‐TK vector (Luciferase Assay System; Promega). Cells were transfected with mimic control, inhibitor control, miR‐556 or antagomir miR‐556 (GenePharma, Shanghai) in different group. Day 3, the luciferase activity of cells was examined on Synergy H1 microplate reader (BioTek Instruments, Winooski, VT, USA) using Dual‐luciferase Reporter Assay System (Promega) according to the instruction of manufactures. Results were represented as relative luciferase activity to pRL‐TK.

### 2.7 Measurement of reactive oxygen species (ROS) activity

ROS activity was measured in ccRCC cells from 17 patients with different rs1055259 genotypes (AA = 8, AG = 6, GG = 3) using DCFH‐DA (Beyotime). It is an oxidation‐sensitive fluorescence probe. Cells were seeded in 6‐well plates (1 × 10^5^) and incubated with DCFH‐DA in 37°C for 20 minutes. After washing with PBS, the nuclei were stained by DAPI (Beyotime). The cells were imaged on a Carl Zeiss LSM880 laser scanning confocal microscope (Carl zeiss, Jena, Germany). Quantitative analysis of DCF fluorescence was conducted with Image Pro Plus 6.0 (Media Cybernetics, Silver Spring, MD, USA).

### 2.8 Cell proliferation and invasion assay

Proliferation of ccRCC cells was determined using cell counting kit‐8 (CCK‐8) (Dojindo Laboratories, Kumamoto, Japan) according to the manufacturer's instructions. Cell invasion measurements were performed with 24‐well Transwell^®^ units (8.0 μm pore; Costar Corning, NY, USA). In brief, cells (1 × 10^3^) in 100 μL were added to each matrigel‐coated insert, and 500 μL of DMEM with 10% FBS was added to the lower chamber, with ROS scavenger N‐acetycysteine (NAC) supplement or not. The plates were incubated at 37°C, 5% CO2 for 24 hours. The non‐invaded cells in the insert were wiped away, and the invaded cells were stained with ponceau. Finally, the images were captured with a phasecontrast microscope (Olympus IX51, Tokyo, Japan). The invaded cells were quantified by counting 10 independent visual fields to determine the average invasion ratio. Each assay was performed in triplicate.

### 2.9 Statistical analysis

The differences of the demographic and clinical features and frequencies of genotypes in case‐control study were calculated by the chi‐square test (for categorical variables) or Student's *t*‐test (for continuous variables). Hardy‐Weinberg equilibrium (HWE) was evaluated by online analytical tools (https://ihg.gsf.de/cgi-bin/hw/hwa1.pl). The linkage disequilibrium (LD) of candidate SNPs was analysed using HaploView v4.2 (Broad Institute, Cambridge, MA, USA). For the determination of main effect of SNPs, univariate and multivariate logistic regression models were conducted to generate odds ratio (ORs) and corresponding 95% confidence intervals (CIs) with adjustment for possible confounders, that is age, gender, drinking status and so on. All statistical tests were 2‐sided and performed with Statistical Program for Social Sciences (SPSS 16.0, Chicago, IL, USA) and R (http://www.r-project.org/). *P *<* *.05 was considered as statistically significant.

## 3 RESULTS

### 3.1 Association of SNPs with ccRCC susceptibility

rs1537236, rs3814309, rs1109138, rs1055259 and rs5776997 were conformed to HWE in healthy controls, with the *P* value of .417, .695, .510, .728 and .315, respectively. Among the 5 candidate SNPs, rs1055259 variant was significantly associated with an decreased susceptibility of ccRCC. Compared with wild‐type AA, AG and AG + GG genotypes were significantly associated with reduced risk of ccRCC (AG vs AA: adjusted OR = 0.61, 95% CI = 0.43‐0.90, *P *=* *.012; AG + GG vs AA: adjusted OR = 0.59, 95% CI = 0.41‐0.92, *P *=* *.019). In allele comparing model, the rs1055259‐G allele was significantly associated with a decreased susceptibility of ccRCC in comparison with A allele (adjusted OR = 0.72, 95% CI = 0.51‐0.98, *P *=* *.040) (Table 3).

**Table 3:** Logistic regression analysis of associations between the genotypes of *GSTM3* and ccRCC susceptibility

Variants	Genotypes	Cases No. (%) n = 329	Controls No. (%) n = 420	Crude OR (95% CI)	*P* a	Adjusted OR (95% CI)	*P* b
rs1537236 (HWE = 0.417)	AA	229 (69.6)	281 (66.9)	1.00 (Reference)		1.00 (Reference)	
	AG	89 (27.0)	122 (29.0)	0.90 (0.65‐1.24)	.511	0.95 (0.72‐1.34)	.693
	GG	11 (3.3)	17 (4.0)	0.79 (0.37‐1.73)	.697	0.86 (0.44‐1.89)	.825
	AG + GG	100 (30.3)	139 (33.0)	0.86 (0.65‐1.20)	.477	0.92 (0.72‐1.35)	.602
	A allele	547 (83.1)	684 (81.4)	1.00 (Reference)		1.00 (Reference)	
	G allele	111 (16.6)	156 (19.6)	0.89 (0.88‐1.16)	.415	0.95 (0.94‐1.23)	.579
rs3814309 (HWE = 0.695)	CC	179 (54.4)	243 (57.9)	1.00 (Reference)		1.00 (Reference)	
	CT	127 (38.6)	151 (36.0)	1.14 (0.84‐1.55)	.436	1.18 (0.89‐1.63)	.522
	TT	23 (7.0)	26 (6.2)	1.20 (0.66‐2.17)	.546	1.35 (0.79‐2.34)	.621
	CT + TT	150 (45.6)	177 (42.2)	1.15 (0.86‐1.54)	.373	1.23 (0.91‐1.79)	.427
	C allele	485 (73.7)	637 (75.8)	1.00 (Reference)		1.00 (Reference)	
	T allele	173 (26.3)	203 (24.2)	1.12 (0.89‐1.42)	.368	1.24 (0.91‐1.47)	.479
rs1109138 (HWE = 0.510)	AA	21 (6.4)	15 (3.6)	1.00 (Reference)		1.00 (Reference)	
	AG	85 (25.8)	118 (28.1)	0.79 (0.48‐1.30)	.370	0.84 (0.56‐1.51)	.491
	GG	223 (67.8)	287 (68.3)	0.86 (0.55‐1.37)	.559	0.91 (0.67‐1.59)	.672
	AG + GG	308 (93.6)	405 (96.4)	0.84 (0.54‐1.31)	.493	0.90 (0.62‐1.56)	.569
	A allele	127 (19.3)	148 (17.6)	1.00 (Reference)		1.00 (Reference)	
	G allele	531 (80.7)	692 (82.4)	0.99 (0.79‐1.26)	1.000	0.99 (0.80‐1.29)	1.000
rs1055259 (HWE = 0.728)	AA	252 (76.6)	289 (68.8)	1.00 (Reference)		1.00 (Reference)	
	AG	68 (20.7)	120 (28.6)	0.65 (0.46‐0.92)	**.014**	0.61 (0.43‐0.90)	**.012**
	GG	9 (2.7)	11 (2.6)	0.94 (0.38‐2.30)	1.000	0.98 (0.45‐2.41)	1.000
	AG + GG	77 (23.4)	131 (31.2)	0.67 (0.49‐0.94)	**.021**	0.59 (0.41‐0.92)	**.019**
	A allele	572 (86.9)	698 (83.1)	1.00 (Reference)		1.00 (Reference)	
	G allele	86 (13.1)	142 (16.9)	0.74 (0.55‐0.99)	**.043**	0.72 (0.51‐0.98)	**.040**
rs5776997 (HWE = 0.315)	‐ ‐	234 (71.1)	317 (75.5)	1.00 (Reference)		1.00 (Reference)	
	‐ T	80 (24.3)	93 (22.1)	1.17 (0.83‐1.64)	.429	1.26 (0.96‐1.73)	.517
	TT	15 (4.6)	10 (2.4)	2.03 (0.90‐4.60)	.099	2.11 (0.93‐4.81)	.126
	‐ T + TT	95 (28.9)	103 (24.5)	1.25 (0.90‐1.73)	.183	1.32 (0.95‐1.89)	.231
	‐ allele	548 (83.3)	727 (86.5)	1.00 (Reference)		1.00 (Reference)	
	T allele	110 (16.7)	113 (13.5)	1.28 (0.96‐1.70)	.107	1.39 (0.99‐1.83)	.198

CI, confidence interval; OR, odds ratio. The significant *P* values were shown in bold.

a Chi‐square test for genotype distributions between cases and controls.

b Adjusted for age, sex, smoking and drinking status in logistic regress models.

### 3.2 Association of SNP rs1055259 genotypes with miR‐556 binding and *GSTM3* expression

Bioinformatical analyses suggested that rs1055259 was located at miR‐556 binding site, the 3′UTR of *GSTM3*. The A allele was predicted to bind more stably with miR‐556 than G allele, but the binding site in mutant sequence was completely destroyed (Figure 2B). To evaluate the different affinity of A and G allele to miR‐556, 3 luciferase reporter constructs were created. In ccRCC cells transfected with rs1055259‐A allele construct, miR‐556 mimic attenuated the luciferase activity by 45.43% while compared with control group; however, miR‐556 mimic only reduced the luciferase activity by 25.61% in rs1055259‐G allele group (Figure 2C). Antagomir‐miR‐556 increased the luciferase activity by 20.14% in A allele group than that by 14.96% in G allele group (Figure 2D). Intriguingly, CoCl2 treatment reduced the luciferase activity by 31.22% in A allele group and 20.59% in G allele group (Figure 2E). Mutant construct losing the miR‐556 binding site did not represent any obvious change. Based on the above evidences, miR‐556 mimic could knock down *GSTM3* expression in both rs1055259‐A and G allele groups. Antagomir‐miR‐556 increased *GSTM3* expression in A and G allele groups. However, among the 3 conditions (control, miR‐556 mimic, antagomir‐miR‐556), cells carrying A allele construct presented lower luciferase activity than those with G allele. Destruction of the miR‐556 binding site interrupted the effects of miR‐556, antagomir‐miR‐556 or CoCl2 on *GSTM3* expression.

**Figure 3:**
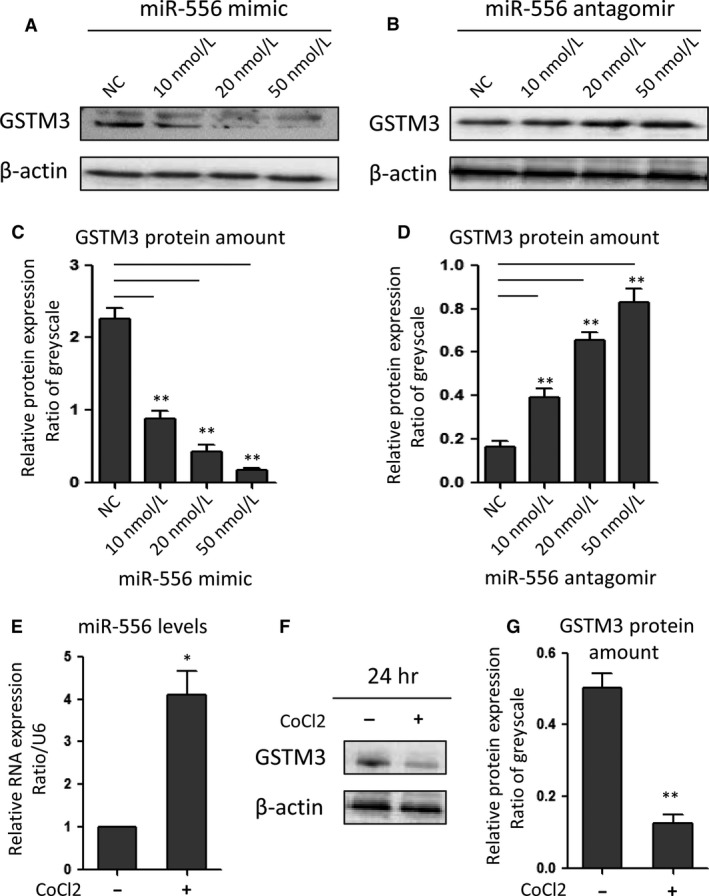
miR‐556 inhibited *GSTM3* expression in 786‐O cells. (A, B) Western blot assay of *GSTM3* in ccRCC cells transfected with miR‐556 mimic or antagomir‐miR‐556 in different doses. (C, D) Greyscale quantification of the *GSTM3* protein expression levels in ccRCC cells transfected with miR‐556 mimic or antagomir‐miR‐556. (E) 50 μM CoCl2 treatment increased miR‐556 mRNA levels. (G, F) Western blot and quantification plot showed that CoCl2 treatment reduced *GSTM3* protein expression. *P <* *.05, **P <* *.01

*GSTM3* expression was further compared across cells transfected with miR‐556 in different dose. The data indicated that miR‐556 mimic dose dependently reduced *GSTM3* expression, and antagomir‐miR‐556 increased *GSTM3* expression (Figure 3A‐D). Compared to the control group, CoCl2 decreased *GSTM3* protein level by 76.75%, with an increased miR‐556 level by 3.03‐fold simultaneously (Figure 3E‐G). For in vitro study, CoCl2 is commonly used to mimic a hypoxic environment in inner area of solid tumor. These data suggested that the malignant progression of renal carcinoma might inhibit the *GSTM3* expression via increasing miR‐556 level.

### 3.3 rs1055259 variant reduced ROS activity

**Figure 4:**
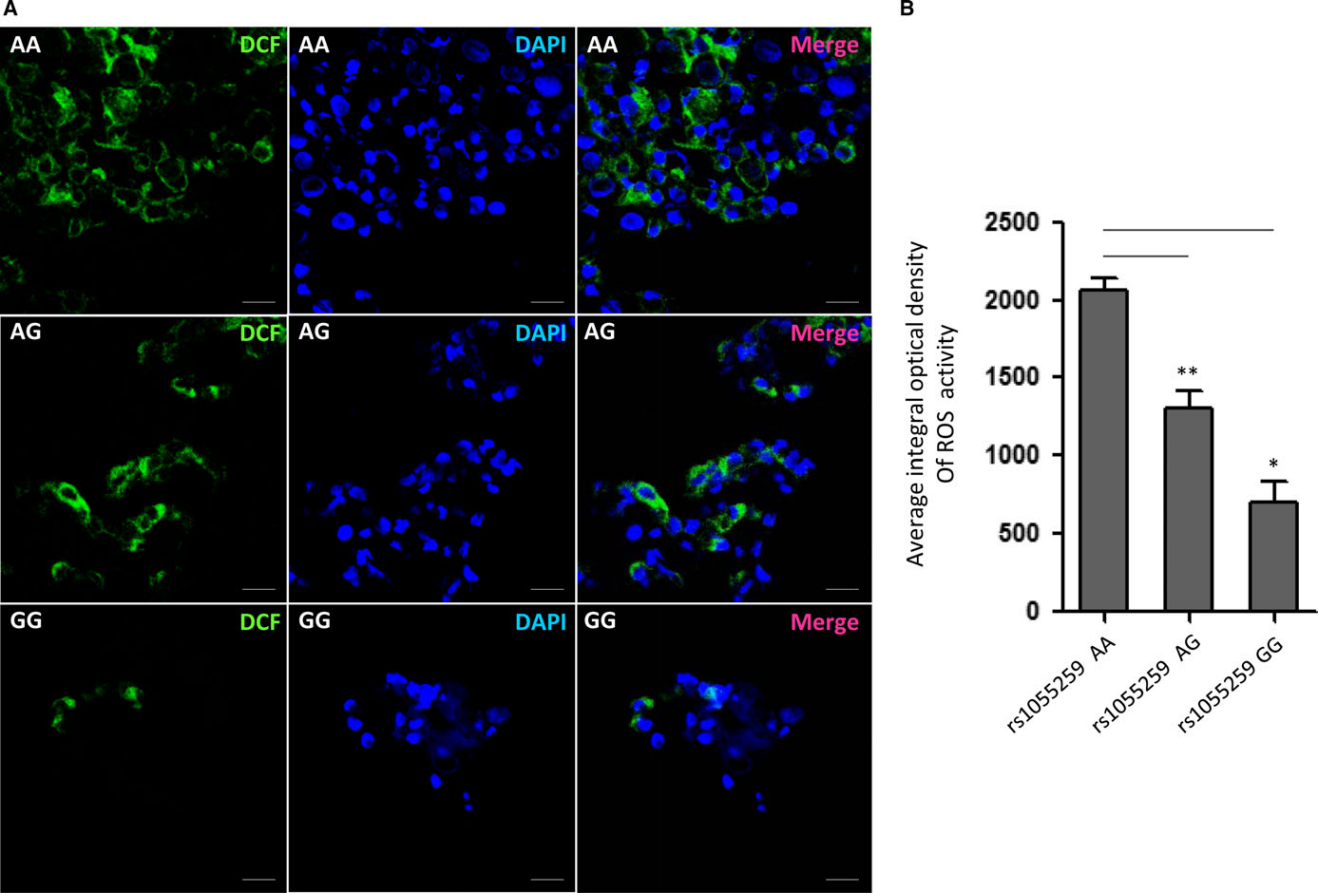
Reactive oxygen species (ROS) activity in ccRCC primary cells with different genotype of rs1055259. (A) Representative confocal‐microscopy graphs of ROS activity in rs1055259 AA, AG and GG cells incubated with DCFH‐DA (it could be ROS‐oxidized to DCF, which presented green fluorescence). (B) Quantitative analysis of ROS activity in different group. The experiment was repeated at least 3 times. Data were presented as mean ± S.D. Scale bar: 50 μm. *P <* *.05, **P <* *.01

To evaluate the biological effect of rs1055259 on *GSTM3* activity in vivo, we measured the ROS activity in ccRCC cells from patients with AA, AG and GG genotypes. As shown in Figure 4, the *GSTM3* activity was obviously different among patients with rs1055259 AA, AG and GG genotypes. The average ROS activity was highest in AA genotype, followed by AG and then lowest in GG genotype (2067.43 ± 117.76, 1310.65 ± 162.07 and 708.51 ± 79.34, respectively). This result suggested that rs1055259 variant, which was related with *GSTM3* expression, indeed reduced ROS activity.

### 3.4 rs1055259 variant suppressed the proliferation and invasion of ccRCC cells

**Figure 5:**
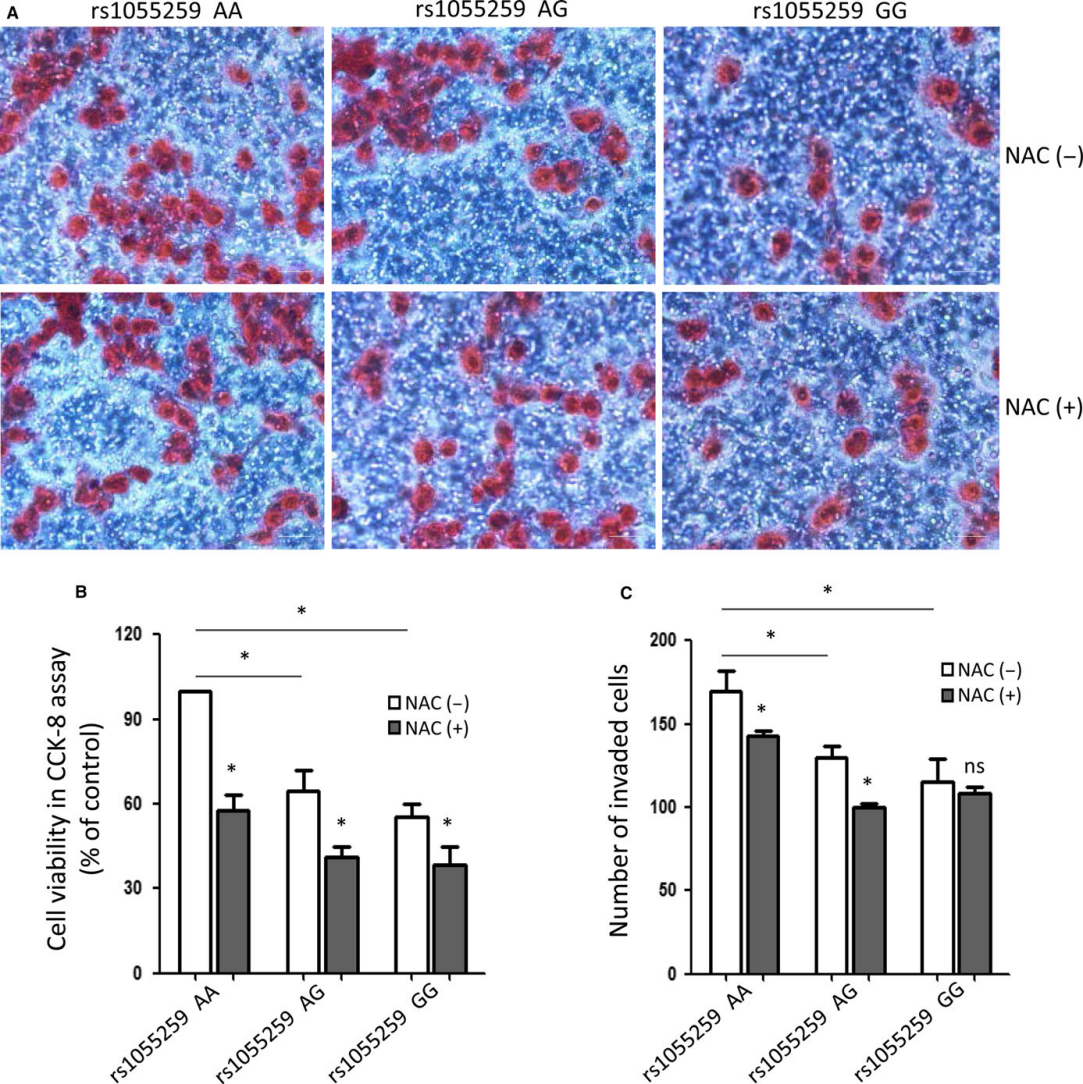
The proliferation and invasion of ccRCC primary cells with different genotype after reactive oxygen species (ROS) scavenger NAC (5 mmol/L) treatment or not. (A) Representative graphs for the invasion assay of rs1055259 AA, AG and GG cells with NAC treatment or not. (B) The percentages of cell viability in CCK‐8 proliferation assay. (C) Quantitative analysis of invasion cells in different groups. The experiment was repeated at least 3 times. Data were presented as mean ± SD. Scale bar: 50 μm. *P <* *.05

It has been proven that *GSTM3* is a tumour suppressor in ccRCC.^8^ To confirm the relationship between rs1055259 and progression of ccRCC, we detected the proliferation and invasion of ccRCC cells with AA, AG and GG genotypes under NAC+/− conditions. The data showed that invasion cells trans‐micropore filtration membranes per visual field were the most in AA group, then AG and GG groups (169.52 ± 17.68, 130.76 ± 9.91 and 115.29 ± 19.80, respectively). While the ROS scavenger‐NAC was added in the concentration of 1 mmol/L, the number of invasion cells was obviously decreased by 18.17% in AA group and 23.12% in AG group, but no significant change in GG group (Figure 5A,C). Similarly, the cell viability for proliferation ability investigation in AA group was the highest (standardized as 100%) than AG and GG groups (64.50% and 55.39%, respectively). In addition to NAC, the cell viability was notably reduced by 43.52%, 36.43% and 30.71% in AA, AG and GG groups, respectively (Figure 5B). Taken together, rs1055259 variant substantially suppressed the proliferation and invasion of ccRCC cells. NAC inhibited the ROS activity and thus repressed the progression of ccRCC cells.

## 4 DISCUSSION

**Figure 6:**
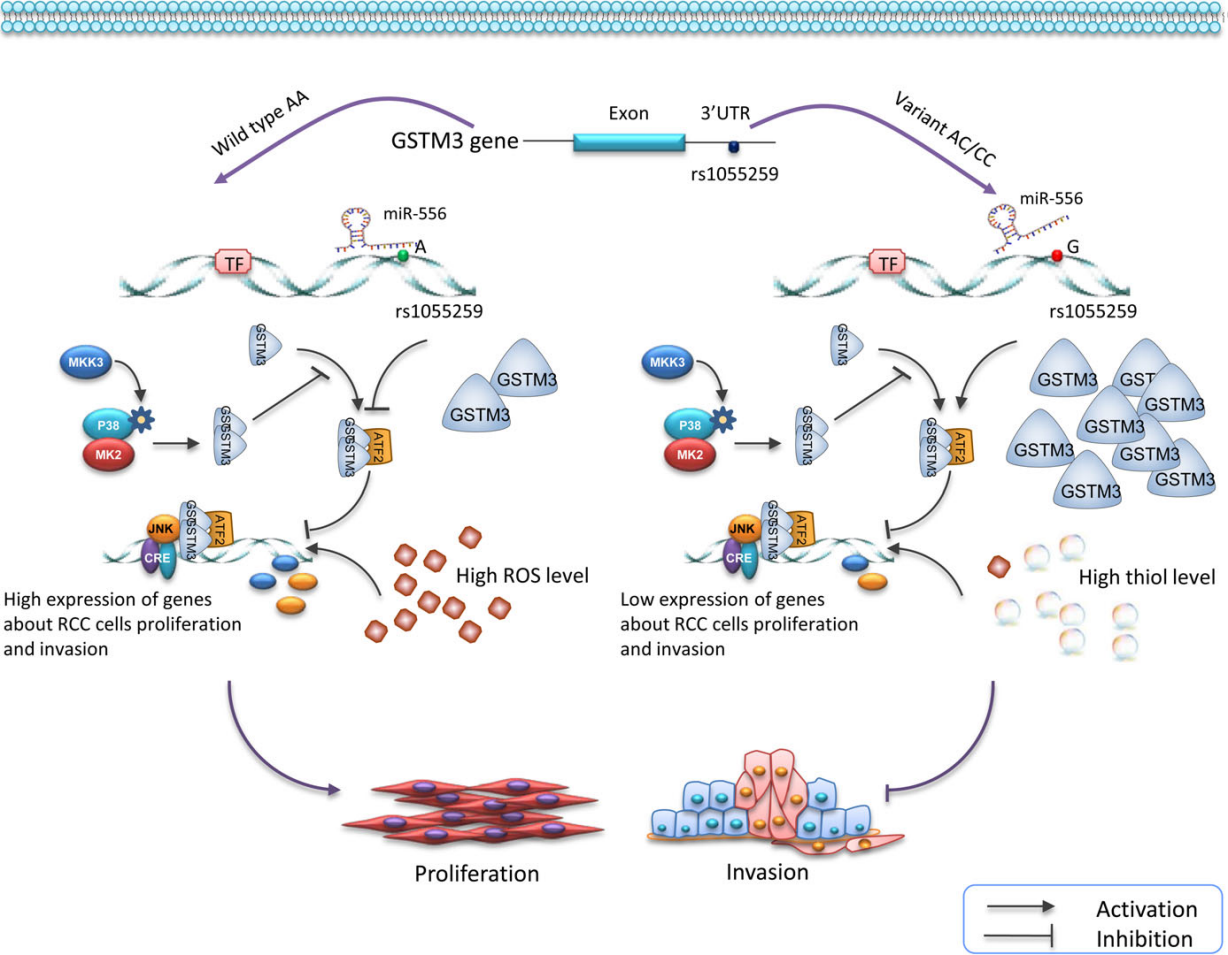
Schematic diagram for the potential functions of rs1055259 in *GSTM3* expression and progression of ccRCC. Variant G allele of rs1055259 might elevate *GSTM3* expression by affecting the binding of miR‐556 and 3′UTR of *GSTM3* gene, resulting in low reactive oxygen species (ROS) level and high thiol level, and further suppress the proliferation and invasion of ccRCC cells

In the present study, we studied the genetic effects of SNPs in 3′UTR of *GSTM3* on the progression of ccRCC. Five candidate SNPs were MALDI‐TOF MS genotyped in 329 ccRCC patients and 420 healthy controls. A significant association between rs1055259 and decreased ccRCC susceptibility was identified. Variant G allele of rs1055259 enhanced *GSTM3* expression via affecting the binding of miR‐556 and 3′UTR, resulting in low ROS level, and further suppressing the proliferation and invasion of ccRCC cells (Figure 6).

rs1055259 is located at the 3′UTR of *GSTM3* and predicted in a miR‐556 binding site, with transcription regulatory potential. When allele A changes to G, the secondary folding structure of mRNA changed (Figure S1); the affinity score of theoretical mRNA: miRNA duplex is strikingly decreased, and the binding site is lost. These analysis data acquired using online bioinformatical software rSNP and SNPinfo confirmed the significance of this locus in transcriptional regulation. According to the evidences shown in Figure 2 and 3, rs1055259 A > G change increased the transcriptional activity, affected the miRNA binding and enhanced *GSTM3* expression. To the best of our knowledge, the functional polymorphism‐rs1055259 has not been reported in other investigations. This is the first study to demonstrate the potential function of rs1055259 in *GSTM3* expression and ccRCC development.

Another intriguing finding was that rs1055259 might be related to ROS activity, by fine tuning the *GSTM3* expression. As is known to all, cytosolic glutathione S‐transferases (GSTs) are members of phase II metabolic isozymes super family. They protect cells against electrophilic damage via catalysing the conjugation of ROS and glutathione.^9^, ^10^ Dysfunction of GSTs has been implicated in the progression of RCC, because of the defect of anti‐oxidant capacity.^11^ Our study showed that the ROS activity (reflected by DCF immunofluorescence) was highest in ccRCC cells with rs1055259 AA genotype than AG and GG genotypes. However, rs1055259 variant was proven associated with *GSTM3* expression. Namely rs1055259 might modulate the ROS activity via elevating *GSTM3* expression. Thus, the subjects carrying A allele are more prone to ccRCC because their ROS activity tends to be higher against those with G allele.

Generous studies revealed that epigenetic inactivation of *GSTM3* or low *GSTM3* expression correlated with tumorigenesis and advanced AJCC stage of cancer.^12^, ^13^ These reports, together with our previously study,^7^ suggested that *GSTM3* might function as a suppressor in tumour progression. Elevated ROS activity also played an important role in cancer cell survival and development.^14^ Thus, we detected the relationship between rs1055259, ROS activity and ccRCC development. In transwell assay, we found that the invasion cells per visual field were the most in AA group and then AG and GG groups. After ROS scavenger‐NAC was added, the numbers of invasion cells were obviously decreased. Similarly, the cell viability in AA group was higher than AG and GG group. With NAC+, the cell viability was notably reduced by more than 30%. Taken together, rs1055259 variant substantially suppressed the proliferation and invasion of ccRCC cells. NAC inhibited the ROS activity and thus repressed the progression of ccRCC cells. It suggested that *GSTM3* might act as a tumour suppressor via ROS activity regulation.

There were several limitations in this study: (1) because all the participants were collected from eastern Han Chinese population, the association between *GSTM3* variants and ccRCC risk might not be generalized to overall ethnic groups. (2) The large cohort, multi‐centre studies about association between rs1055259 and ccRCC risk should be performed to generate more potent statistical powers. (3) ccRCC is a complicated process. The genetic variation and the effects of tumour environmental cues such as oxygen variability might contribute to the tumour heterogeneity and development. The producing and neutralizing of ROS are pivotal in tumour development because many factors, lipids and nucleic acids are downstream targets of ROS.^15^, ^16^, ^17^ Several studies revealed that unregulated and augmented ROS could initiate redox‐linked signalling responses and irreversible injuries in RCC.^18^, ^19^ Moreover, changes of cellular redox balance in RCC might also be associated with alterations of glutathione S‐transferase (GST) expression and phenotype.^18^ Therefore, it is necessary to clarify underlying mechanisms between the important anti‐oxidant enzyme‐GSTM3 in ccRCC,^7^, ^8^ thiol level, ROS activity and redox signalling responses. The further investigations should be carried out.

In summary, we demonstrated that rs1055259 in *GSTM3* 3′UTR affected the miR‐556 binding and *GSTM3* expression in ccRCC for the first time. The G allele of rs1055259 predisposes hosts to down‐regulated *GSTM3* and reduced ccRCC susceptibility. rs1055259 variant genotype was a significant genetic protect factor. These findings will be helpful in diagnosis prediction and surveillance of ccRCC, as well as in the advancement of targeted therapy for this renal malignancy. The current study also indicated that it warranted further investigations of *GSTM3* expression in ccRCC.

## CONFLICTS OF INTEREST

The authors have declared that no conflicts of interest exist.

## AUTHOR CONTRIBUTIONS

Y.W., F.L., H.S. and Z.S. designed the experiments and analysed the data. Y.W., Z.Y., H.H. and H.S. performed all the experiments. Y.W. and Y.C. wrote the manuscript and prepared the figures. All authors reviewed and approved the final manuscript.

## References

[1] Chow WH , Dong LM , Devesa SS . Epidemiology and risk factors for kidney cancer. Nat Rev Urol. 2010;7:245‐257. 20448658 10.1038/nrurol.2010.46 PMC3012455

[2] Angevin E , Lopez‐Martin JA , Lin CC , et al. Phase I study of dovitinib (TKI258), an oral FGFR, VEGFR, and PDGFR inhibitor, in advanced or metastatic renal cell carcinoma. Clin Cancer Res. 2013;19:1257‐1268. 23339124 10.1158/1078-0432.CCR-12-2885

[3] Purdue MP , Johansson M , Zelenika D , et al. Genome‐wide association study of renal cell carcinoma identifies two susceptibility loci on 2p21 and 11q13.3. Nat Genet. 2011;43:60‐65. 21131975 10.1038/ng.723 PMC3049257

[4] Schodel J , Bardella C , Sciesielski LK , et al. Common genetic variants at the 11q13.3 renal cancer susceptibility locus influence binding of HIF to an enhancer of cyclin D1 expression. Nat Genet. 2012;44:420‐425, S1–2. 22406644 10.1038/ng.2204 PMC3378637

[5] Wu X , Scelo G , Purdue MP , et al. A genome‐wide association study identifies a novel susceptibility locus for renal cell carcinoma on 12p11.23. Hum Mol Genet. 2012;21:456‐462. 22010048 10.1093/hmg/ddr479 PMC3276284

[6] Su T , Han Y , Yu Y , et al. A GWAS‐identified susceptibility locus on chromosome 11q13.3 and its putative molecular target for prediction of postoperative prognosis of human renal cell carcinoma. Oncol Lett. 2013;6:421‐426. 24137339 10.3892/ol.2013.1422 PMC3789013

[7] Tan X , Zhai Y , Chang W , et al. Global analysis of metastasis‐associated gene expression in primary cultures from clinical specimens of clear‐cell renal‐cell carcinoma. Int J Cancer. 2008;123:1080‐1088. 18546293 10.1002/ijc.23637

[8] Tan X , Wang Y , Han Y , et al. Genetic variation in the GSTM3 promoter confer risk and prognosis of renal cell carcinoma by reducing gene expression. Br J Cancer. 2013;109:3105‐3115. 24157827 10.1038/bjc.2013.669 PMC3859948

[9] Strange RC , Spiteri MA , Ramachandran S , Fryer AA . Glutathione‐S‐transferase family of enzymes. Mutat Res. 2001;482:21‐26. 11535245 10.1016/s0027-5107(01)00206-8

[10] Dusinska M , Staruchova M , Horska A , et al. Are glutathione S transferases involved in DNA damage signalling? Interactions with DNA damage and repair revealed from molecular epidemiology studies. Mutat Res. 2012;736:130‐137. 22450146 10.1016/j.mrfmmm.2012.03.003

[11] Sweeney C , Farrow DC , Schwartz SM , Eaton DL , Checkoway H , Vaughan TL . Glutathione S‐transferase M1, T1, and P1 polymorphisms as risk factors for renal cell carcinoma: a case‐control study. Cancer Epidemiol Biomarkers Prev. 2000;9:449‐454. 10794492

[12] Peng DF , Razvi M , Chen H , et al. DNA hypermethylation regulates the expression of members of the Mu‐class glutathione S‐transferases and glutathione peroxidases in Barrett's adenocarcinoma. Gut. 2009;58:5‐15. 18664505 10.1136/gut.2007.146290 PMC2845391

[13] Lim R , Lappas M , Ahmed N , Permezel M , Quinn MA , Rice GE . 2D‐PAGE of ovarian cancer: analysis of soluble and insoluble fractions using medium‐range immobilized pH gradients. Biochem Biophys Res Commun. 2011;406:408‐413. 21329656 10.1016/j.bbrc.2011.02.056

[14] Zhang C , Cao S , Toole BP , Xu Y . Cancer may be a pathway to cell survival under persistent hypoxia and elevated ROS: a model for solid‐cancer initiation and early development. Int J Cancer. 2015;136:2001‐2011. 24828886 10.1002/ijc.28975

[15] Block K , Gorin Y , New DD , et al. The NADPH oxidase subunit p22phox inhibits the function of the tumor suppressor protein tuberin. Am J Pathol. 2010;176:2447‐2455. 20304964 10.2353/ajpath.2010.090606 PMC2861109

[16] Block K , Gorin Y . Aiding and abetting roles of NOX oxidases in cellular transformation. Nat Rev Cancer. 2012;12:627‐637. 22918415 10.1038/nrc3339 PMC3711509

[17] Ralph SJ , Rodriguez‐Enriquez S , Neuzil J , Saavedra E , Moreno‐Sanchez R . The causes of cancer revisited: “mitochondrial malignancy” and ROS‐induced oncogenic transformation – why mitochondria are targets for cancer therapy. Mol Aspects Med. 2010;31:145‐170. 20206201 10.1016/j.mam.2010.02.008

[18] Pljesa‐Ercegovac M , Mimic‐Oka J , Dragicevic D , et al. Altered antioxidant capacity in human renal cell carcinoma: role of glutathione associated enzymes. Urol Oncol. 2008;26:175‐181. 18312938 10.1016/j.urolonc.2007.02.007

[19] Lusini L , Tripodi SA , Rossi R , et al. Altered glutathione anti‐oxidant metabolism during tumor progression in human renal‐cell carcinoma. Int J Cancer. 2001;91:55‐59. 11149420 10.1002/1097-0215(20010101)91:1<55::aid-ijc1006>3.0.co;2-4

