# Supplementary material for: A novel functional polymorphism of GSTM3 reduces clear cell renal cell carcinoma risk through enhancing its expression by interfering miR‐556 binding
Source: J Cell Mol Med. 2018 Mar 22;22(6):3005–15. doi: 10.1111/jcmm.13528 (PMC5980204; doi:10.1111/jcmm.13528)
Supplement: Supplementary file 1 [file JCMM-22-3005-s001.docx]

**A novel functional polymorphism of *GSTM3* reduces clear cell renal cell carcinoma risk through enhancing its expression** **by interfering miR-556 binding**

Ying Wang^1^, Feng Li^2^, Yi-Huan Chen^1^, Han Shen^1^, You Yu^1^, Zhen-Ya Shen^1^*

^1^Department of Cardiovascular Surgery, First Affiliated Hospital & Institute for Cardiovascular Science, Soochow University, Suzhou, Jiangsu, China

^2^Department of Urinary Surgery, First Affiliated Hospital of Soochow University, Suzhou, Jiangsu, China

*Correspondence to: Zhen-Ya Shen, Department of Cardiovascular Surgery, First Affiliated Hospital& Institute for Cardiovascular Science, Soochow University, Suzhou, China. Email: uushen@aliyun.com, Fax/Tel:0512-67972066.


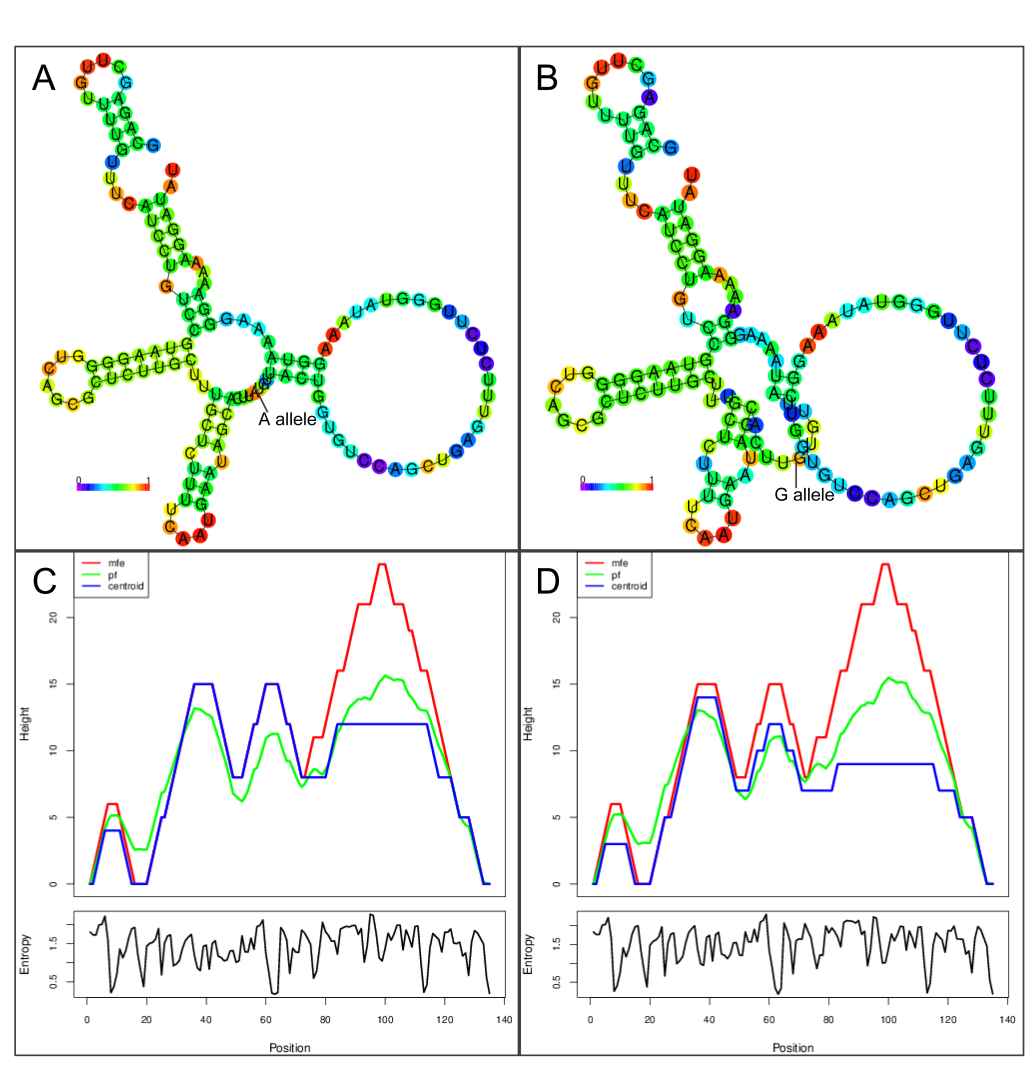


Supplemental Fig 1. Bioinformatic prediction of rs1055259 impact on RNA folding structures of *GSTM3* 3'UTR. The structures corresponding to rs1055259 A (A) or G allele (B). Mountain plots related to rs1055259 A (C) or G allele (D). y-axis: minimum free energy or entropy. x-axis: sequence position.
